# Isolation and characterization of a β-propeller gene containing phosphobacterium *Bacillus subtilis* strain KPS-11 for growth promotion of potato (*Solanum tuberosum* L.)

**DOI:** 10.3389/fmicb.2015.00583

**Published:** 2015-06-09

**Authors:** Muhammad Kashif Hanif, Sohail Hameed, Asma Imran, Tahir Naqqash, Muhammad Shahid, Jan D. Van Elsas

**Affiliations:** ^1^Microbial Physiology Lab, Department of Microbial Ecology, Soil and Environmental Biotechnology Division, National Institute for Biotechnology and Genetic EngineeringFaisalabad, Pakistan; ^2^Department of Bioinformatics and Biotechnology, Government College UniversityFaisalabad, Pakistan; ^3^Department of Microbial Ecology, Centre for Ecological and Evolutionary Studies, University of GroningenGroningen, Netherlands

**Keywords:** phosphobacteria, β-propeller, *Bacillus*, potato, growth promotion

## Abstract

Phosphate-solubilizing and phytate-mineralizing bacteria collectively termed as phosphobacteria provide a sustainable approach for managing P-deficiency in agricultural soils by supplying inexpensive phosphate to plants. A phosphobacterium *Bacillus subtilis* strain KPS-11 (Genbank accession no. KP006655) was isolated from potato (*Solanum tuberosum* L.) rhizosphere and characterized for potato plant growth promoting potential. The strain utilized both Ca-phosphate and Na-phytate *in vitro* and produced 6.48 μg mL^-1^ indole-3-acetic acid in tryptophan supplemented medium. P-solubilization after 240 h was 66.4 μg mL^-1^ alongwith the production of 19.3 μg mL^-1^ gluconic acid and 5.3 μg mL^-1^ malic acid. The extracellular phytase activity was higher (4.3 × 10^-10^ kat mg^-1^ protein) than the cell-associated phytase activity (1.6 × 10^-10^ kat mg^-1^ protein). *B. subtilis* strain KPS-11 utilized 40 carbon sources and showed resistance against 20 chemicals in GENIII micro-plate system demonstrating its metabolic potential. Phytase-encoding gene β-propeller (BPP) showed 92% amino acid similarity to BPP from *B. subtilis* (accession no.WP_014114128.1) and 83% structural similarity to BPP from *B. subtilis* (accession no 3AMR_A). Potato inoculation with *B. subtilis* strain KPS-11 increased the root/shoot length and root/shoot weight of potato as compared to non-inoculated control plants. Moreover, rifampicin-resistant derivative of KPS-11 were able to survive in the rhizosphere and on the roots of potato up to 60 days showing its colonization potential. The study indicates that *B. subtilis* strain KPS-11 can be a potential candidate for development of potato inoculum in P-deficient soils.

## Introduction

Phosphorous (P) ranks second among essential plant nutrients and a major component of vital molecules such as nucleic acid, ATP and phospholipids. It is involved in regulating cell metabolic activity by controlling key enzymatic reactions (Schachtman et al., 1998; Collavino et al., 2010; Gupta et al., 2012). Most of the soils contain a substantial amount of P but its bioavailability is usually low due to its low solubility and rapid conversion to insoluble forms (Jorquera et al., 2011). P-fertilizers readily become insoluble due to the formation of precipitates with iron and aluminum ions. Furthermore, in acidic soils, P is adsorbed with clay particles or organic matter while in alkaline soils, it is converted to calcium phosphates (Mullen, 2005; Goldstein and Krishnaraj, 2007).

Plant rhizosphere harbors a variety of microorganisms that can significantly change the soil P dynamics. These microorganisms collectively called as phosphobacteria are involved either in solubilizing the inorganic forms of phosphates by producing organic acid e.g., gluconic, 2-ketogluconic acid, malic, lactic, acetic, citric, and succinic acid (Rodríguez et al., 2006; Bianco and Defez, 2010; Shahid et al., 2012) or in mineralization of organic forms of phosphates by enzymatic degradation (Jorquera et al., 2008b). Mineral phosphate solubilization is a complex phenomena which is facilitated by low molecular weight organic acids, through their hydroxyl and carboxyl groups chelate cation bound to phosphate thus converting it to soluble forms (Chen et al., 2006; Vyas and Gulati, 2009; Lavania and Nautiyal, 2013). A gene pqq encods pyrroloquinoline quinine (PQQ) which is a co-factor of holenzyme glucose dehydrogenase (GDH). GDH is responsible for direct extracellular oxidation of glucose into gluconic acid (Perez et al., 2007) resulting in the solubilization of the poorly soluble calcium phosphate. In addition to this, PQQ has been reported as plant growth promoting agent (Choi et al., 2008). In most of the bacteria containing PQQ there are present six or seven genes (pqqABCDEF/G) in an operon. PQQ biosynthesis involves several enzymes encoded by these genes including the pqqE that encodes a regulatory enzyme named as PQQ synthase (Goldstein et al., 2003).

Almost 30–65% of total P in soil is in the form of organic phosphates (Lim et al., 2007) of which phytate constitute the major organic source. The enzyme phytase (*myo*-inositol hexakisphosphate phosphohydrolases) is involved in catalyzing the sequential release of inorganic orthophosphates from phytate (Rodríguez et al., 2006; Jorquera et al., 2008a) resulting in the release of P. Up to 60% of the total organic P may be hydrolyzed by phosphatases of which maximum is released by phytases (Bünemann, 2008). On the basis of structure and variations in their catalytic mechanism, phytases have been grouped as cysteine phytases (CPhy), histidine acid phosphatases (HAP), purple acid phosphatases (PAP), and β-propeller phytases (BPP) (Mullaney et al., 2007). β-propeller phytases has special importance because of their universal occurrence in various rhizosphere soil bacteria and significance for phytate-degradation (Lim et al., 2007; Huang et al., 2009; Menezes-Blackburn et al., 2014).

The rhizobacteria with the ability of both phosphate-solubilization and phytate-mineralization are of great interest for crop improvement, soil management and biotechnological applications. They increase the level of soluble P in soil and its uptake by plants (Igual et al., 2001; Konietzny and Greiner, 2004; Unno et al., 2005; Richardson and Simpson, 2011). The members of genus *Bacillus* are ubiquitous in the rhizosphere and have great potential to be used as inoculum in agriculture or enzyme industry. The *Bacillus* species contribute significantly to plant health due to their multifunctional biofertilization properties i.e., N_2_ fixation, siderophores production, P-solubilization, IAA production and/or antagonistic properties (antibiosis, secretion of lytic enzymes, and induction of systemic resistance in host plant (Ali et al., 2014). Numerous *Bacillus*-based inoculants have already been developed (McSpadden Gardener, 2004) and can carry traits such as phytase production (Jorquera et al., 2008b), or organic acid production (Rodríguez et al., 2006), phytohormone production and/or strains having biocontrol potential (Ali et al., 2014). Surprisingly, few studies have evaluated the ecology, genomics, and the role of *Bacillus* spp. having dual feature of P-solubilization and phytate mineralization in agricultural soils especially from potato rhizosphere.

In this study, we have described the occurrence of phytase-producing *Bacillus* in soils from central Pujnab, Pakistan that typically is well known potato growing region. Potato being the most important non-grain food is popular among both developed and developing world and being used as staple crop in around 130 countries (Calvo et al., 2010). The potato plant is very responsive to P fertilizer and high fertilization is usually recommended to increase early leaf development, tuber setting, quality and yield (Ekelöf, 2007). According to Food and Agriculture Organization (FAO, Current world fertilizer trends and outlook to 2016) South Asia shares around 33% of the total demand for phosphorus based fertilizers in the world, on the other hand it can supply only 3% of total increase in phosphoric acid (as P_2_O_5_). Demand-supply gap, increasing fertilizer cost and fixation of phosphate fertilizers are major constraints for low potato yield in the region especially Pakistan. Considering these issues in mind, a study was planned to characterize rhizobacteria from potato growing areas of central Punjab, Pakistan specifically focusing on their ability to solubilize inorganic phosphates as well as mineralize organic phosphates. Additionally, genes involved in P-solubilization and mineralization were sequenced and bacteria were identified by sequence analysis of 16S rRNA gene. The ultimate aim was to develop eco-friendly P-biofertilizer for sustainable potato growth with low-input and high yield.

## Materials and Methods

### Soil Sampling and Isolation of Phosphobacteria

Samples were collected from potato rhizosphere soil (EC 1.85 dS m^-1^, pH 8, organic matter 0.78%, total N 0.065% and available P 3.25 mg kg^-1^) at 0–15 cm depth from farmer potato field in Jhang (31.27°N, 72.32°E, 141 feet elevation) Pakistan. Samples were kept at 4°C in sterile bottle and immediately transported to the laboratory. For bacterial isolation one gram of rhizosphere soil (in triplicate) was suspended in 9 ml of sterile saline solution (8.9 g L^-1^of NaCl) and vigorously shaken for 10 min and subsequent serial dilution were prepared as described by Somasegaran and Hoben (1994) and spread onto Luria-Bertani (LB) agar medium. After incubation for 48 h at 28 ± 2°C, plates containing an adequate number of colony-forming units (CFUs) were chosen randomly and transferred to screening media used to detect phosphate solubilizing bacteria (PSB) and phytate mineralizing bacteria (PMB). The media used in this study were Pikovskaya’s agar containing tri-calcium phosphate (Pikovskaya, 1948); and phytate-screening medium containing Na-phytate (Kerovuo et al., 1998). The pH of the media was adjusted to 7 and the ability to utilize P on specific media was examined after incubation for 7–10 days at 28 ± 2°C. The appearance of halo-zones around the colonies was taken as an indication of phosphate solubilization and phytate mineralization. Among several bacteria screened, bacterial strain KPS-11 showed the capacity of both phosphate-solubilization and phytate-mineralization, hence selected for detailed studies.

### Strain Identification

Genomic DNA was used to amplify the 16S rRNA gene with primers 27F and 1492R as described Rochelle et al. (1992) with modifications as: for 50 μL reaction in purified water, 10X Taq buffer (Fermentas), 2.5 mM MgCl_2_ (Fermentas), 0.2 mM dNTPs (Fermentas), 10% DMSO, 10 pM each forward and reverse primer, 5 U Taq DNA polymerase (Fermentas) and 40 ng of template DNA. Polymerase chain reaction was carried out in thermal cycler (PeQLab, advanced Primus 96) and temperature conditions were also modified as 30 cycles of 95°C for 2 min, 55°C for 30 s, and 72°C for 5 min. The initial denaturation and final extension steps were 95°C for 5 min and 72°C for 10 min, respectively. Amplified PCR product was purified using Wizard^®^ SV Gel and PCR clean-up system (Promega, USA), and sequenced by Macrogen Korea. The gene sequence was analyzed using sequence scanner software package; compared with others in the Genbank database using the NCBI BLAST (Altschul et al., 1990).

### Phenotypic Microarray

Fresh culture of bacterial strain KPS-11 was grown on LB agar medium and suspended in an ‘Inoculation Fluid’ (IF) purchased from Biolog along with GEN III MicroPlate panel to analyze bacterial cultures for 71 different kind of carbon utilization assays and 23 chemical sensitivity assays. The cell suspension after getting recommended cell density (according to manufacturer) inoculated into the GEN III MicroPlate and each well of MicroPlate was filled with 100 μl using multichannel pipette, was incubated at 28 ± 2°C for 48 h to allow the phenotypic fingerprint to form. After incubation MicroPlates were analyzed qualitatively for color development on VERSA max micro-plate reader with softmax pro-software (Molecular Devices, USA).

### Detection of Indole-3-Acetic Acid (IAA)

Single colony of bacterial strain KPS-11 from pure culture was inoculated to 100 mL LB broth (10 g L^-1^ D-glucose, 5 g L^-1^ yeast extract, 10 g L^-1^ tryptone, 5 g L^-1^ NaCl) in 500 mL Erlenmeyer flasks that was supplemented with L-tryptophan (100 mg L^-1^) as a precursor of IAA biosynthesis. Culture was grown at 28 ± 2°C for 48 h with constant shaking (150 rpm). The cell-free supernatant of culture was obtained by centrifuging the culture at 13,000 × *g* for 10 min, acidified up to pH 2.8 with hydrochloric acid (HCl) and extracted thrice with equal volumes of ethyl acetate (Tien et al., 1979). The extract was evaporated to dryness, collected in 1 mL of methanol and filtered with 0.22 μm nylon filter. Final extract was analyzed on high performance liquid chromatograph (HPLC; Varian pro-star 210/215) at λ = 260 nm and C-18 column using methanol: acetic acid: water (30:1:70 v/v/v) as mobile phase at a flow rate of 1 mL min^-1^.

### Phosphate Solubilization and Detection of Organic Acids

The strain KPS-11 was inoculated in 100 mL of Pikovskaya’s broth in 500 mL flasks in triplicate and incubated in an orbital shaker at 150 rpm at 28 ± 2°C for up to 10 days (240 h). Twenty micro liter of bacterial culture from each flask was harvested at 5, 7, and 10 days post inoculation, centrifuged at 13,000 × *g* for 10 min and cell-free supernatant was collected. Phosphate solubilization was determined through Phospho-molybdate blue color method using spectrophotometer (λ = 882 nm) as described by Murphy and Riley (1962). For HPLC analysis, the cell-free supernatant was filtered through 0.2 μm nylon filters (Millipore, USA) and 20 μL was injected to HPLC equipped with Turbochrom software (Perkin Elmer, USA) and C-18 column at a flow rate of 0.6 mL min^-1^ using 30:1:70 (v/v/v) methanol: acetic acid: water as mobile phase. Signals were detected at 210 nm. The organic acids gluconic, malic, lactic, oxalic, tartaric, and ascorbic acid (Sigma–Aldrich) were used as standard.

### Detection of Phytase Activity

Bacterial strain KPS-11 was grown in a Phytase-screening Medium (PSM) broth at 28 ± 2°C for 48 h. Supernatant was separated by centrifugation at 12,000 × *g* for 5 min and was subjected to ammonium sulfate precipitation, The cell pellets were treated with lysozyme (5 mg mL^-1^) and sonicated (20 kHz constant frequency for 2 min) to break the cells. The cell debris was removed by centrifugation (2300 × *g* for 5 min) and the supernatant was again subjected to ammonium sulfate precipitation. Both pellets, obtained after ammonium sulfate precipitation, were resuspended in Tris-HCl buffer (pH 7.0) and were stored at -20°C as described by Hill et al. (2007). Phytase activity was assayed using the ferrous sulfate- molybdenum blue method (Huang et al., 2006). Briefly, 50 μl of enzyme solution was added in 950 μl substrate solution (1.5 mM sodium phytate in 100 mM Tris–HCl buffer containing 1 mM Ca^2+^, pH 7.0) and incubated at 37°C for 30 min. The reaction was stopped by adding 1 ml 10% (w/v) tri-chloro acetic acid. The released inorganic phosphate was quantified by comparison to a standard curve generated with inorganic P after adding 2 ml coloring reagent C (1% (w/v) ammonium molybdate, 3.2% (v/v) sulfuric acid, and 7.2% (w/v) ferrous sulfate), and the absorbance was measured at 700 nm. Blanks were set by adding the stop solution before sample addition. All phytase activity determinations were performed in triplicate and mean was calculated.

### Analysis of Phytase-Encoding β-Propeller Gene

The genomic DNA of the strain KPS-11 was used in a touchdown PCR using prime primers BPP-F and BPP-R as described by Huang et al. (2009). With some modifications, the template DNA 40 ng was added to a 35 μL reaction mixture containing 5 U Taq polymerase (Bioline), 10X Taq buffer (Fermentas), 2.5 mM MgCl_2_ (Fermentas), 0.2 mM dNTPs (Fermentas), 10% DMSO and 40 pM each forward and reverse primer in MiliQ water. The optimized PCR conditions for primers BPP-F and BPP-R were 4 min at 95°C, followed by eight cycles of 95°C for 30 s, 57°C (decreasing by 1°C after each cycle) for 30 s, and 72°C for 30 s, followed by 27 cycles of 95°C for 30 s, 48°C for 30 s, and 72°C for 30 s and then a final extension at 72°C for 5 min. The presence and size of amplified product was determined through gel electrophoresis; purified using Wizard^®^ SV Gel and PCR clean-up system (Promega, USA) and sequenced by LGC, Germany. The gene sequence was analyzed using sequence scanner software package; compared with other phytase encoding gene sequences in the Genebank database using the NCBI BLAST (Altschul et al., 1990) program for translated nucleotides. Multiple sequences alignment was performed using the Clustal W program (Thompson et al., 1994), and phylogeny of multiple microbial phytases was determined by neighbor-joining method using MEGA6 software with its reliability assessed by 1000 bootstrap repetitions (Tamura et al., 2013). The Molecular Modeling Database (MMDB) from NCBI combined with the software Cn3D was used to visualize the 3D structure of phytase encoding gene BPP (Wang et al., 2007). The presence of signal peptide within phytase gene was checked using signalP 4.0 (Petersen et al., 2011).

### Developing Rifampicin-Resistant Derivatives of KPS-11

Derivatives of strain KPS-11 resistant to rifampicin (antibiotic) were developed by spreading 100 μL of overnight grown culture on LB-agar plates containing 20, 50, 70, and 100 μg mL^-1^ rifampicin. The plates were incubated at 28 ± 2°C for 24 h and the colonies (2–6 plate^-1^) obtained were further streaked on LB-agar plates containing 20, 50, 70, 100 μg mL^-1^ rifampicin. The vigor and suitability of derivatives were checked and compared to the wild type strain by growing both wild type and rifampicin-resistant derivative strains in LB broth at 28 ± 2°C for 24 h. The cultures were harvested after 30 min, 4, 16, and 24 h each, serially diluted and streaked onto LB agar plates. The comparative growth of derivative and wild type strain was estimated by counting CFU mL^-1^ for each interval on a colony counter. The data obtained was converted to log values for both the strains.

### Green House Potato Inoculation Experiment

The objective of the experiment was to evaluate the colonization and phosphate mineralization potential of strain KPS-11 and its effect on potato growth. The experiment was conducted in green house (day/night temperature 25/20°C, light/dark periods 14/10) in 24 cm diameter pots containing 1 kg natural soil with low phosphorous contents (available K: 78.2 kg/ha, available P: 2.6 kg/ha, total N: 9.7 kg/ha, organic matter 2.6% and pH 6.2) mixed with 10 mL 100 g^-1^ inoculum of derivative strain 30 min before tuber sowing. Pre-germinated tubers of cultivar ‘Innovator’ were sowed in pots after dipping in inoculum for 10 min. Plants were watered alternatively with Hoagland’s solution (without P source) and autoclaved distilled water (150 mL pot^-1^) after every 48 h. There were 36 pots in total. Twelve pots (three each for un-inoculated control with and without Na-phytate and inoculated treatments with and without Na-phytate) were harvested at 20, 40, and 60 days after sowing (DAS) and data of growth parameters and bacterial population in rhizosphere and rhizoplane was recorded. Bacterial strain KPS-11 was recovered from rhizosphere and rhizoplane on LB-rifampicin (70 μg mL^-1^) plates by carefully uprooting potato plants with intact roots and shaking gently in sterile distilled water to remove the loosely adhered soil. Bacteria were recovered by dilution plating technique using one gram of strictly adhering soil in 9 mL sterile water for rhizosphere population. After 10 times vigorous shaking with sterile distilled water, 1 g of macerated roots was vigorously vortexed in 9 mL sterile water to remove the root adhering bacteria which are considered as rhizoplane population (Seldin et al., 1998).

### Statistical Analysis

Regression and correlation analyses were performed to determine the relationship among change in pH of medium, time of incubation, soluble P and organic acid production using SPSS software package version 17.0 (SPSS, Inc., Chicago, IL, USA). Data regarding pot experiment was statistically analyzed by analysis of variance technique (Steel et al., 1997), using the Statistix 8.1 software (Tallahassee, FL, USA), and to compare the difference among treatments least significant difference (LSD) test at 5% probability was used.

## Results

### Identification and Metabolic Characterization of Bacterial Strain KPS-11

The bacterial strain KPS-11 isolated from potato rhizosphere was a Gram positive, motile bacterium having rod shaped cell morphology. On LB-agar plates, it showed creamy to brownish colored colonies with irregular margins. The sequence analysis of 16S rRNA gene showed 99% homology to *Bacillus subtilis* strain EB41 (JX683721), *B. subtilis* subsp. *inaquosorum* strain YN32 (KC511536) and *B. subtilis* strain AVS1 (KM110978). On the bases of these sequence similarity results, the strain KPS-11 was named as *B. subtilis* and 16S rRNA gene sequence was deposited to NCBI Genbank under accession number KP006655.

The *B. subtilis* strain KPS-11 was able to produce 6.48 μg mL^-1^ IAA; a known plant growth regulating hormone. Phenotypic microarray analyses done using BIOLOG GNIII micro plates system showed that it utilized 40 different carbon sources, and showed resistance against 20 chemicals except 8% NaCl, D-Serine and Sodium Bromate.

### Inorganic Phosphate Solubilization

**FIGURE 1:**
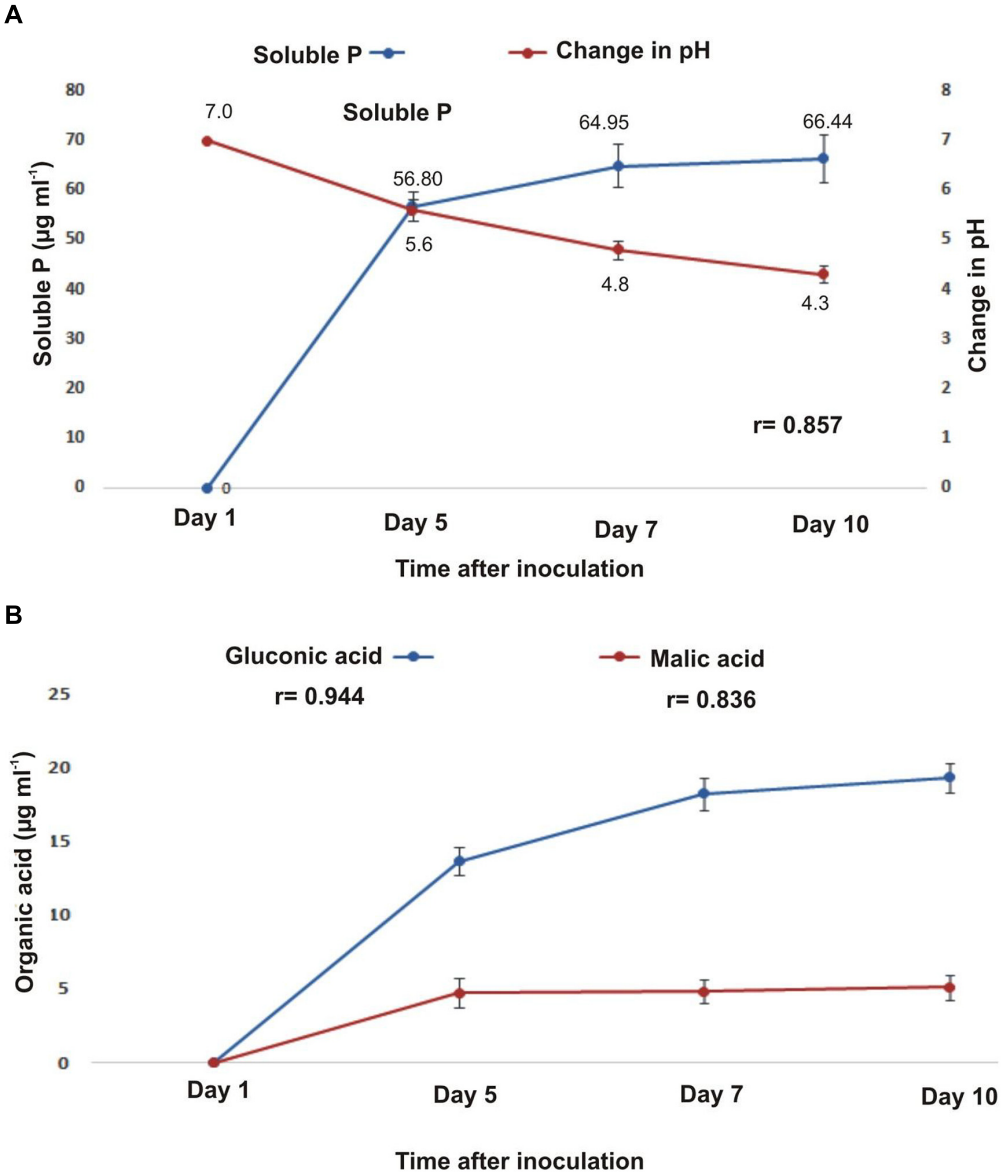
**(A)** Relationship between Phosphorous (P) solubilization (μg mL^-1^) and change in pH in Pikovskaya’s broth with time **(B)**. Detection of organic acids (gluconic acid and malic acid) in *Bacillus subtilis* strain KPS-11 by HPLC at different time intervals (μg mL^-1^). Error bars represent SD (*n* = 3).

*B. subtilis* strain KPS-11 formed halo zone on Pikoviskaya’s agar plates containing tri-calcium phosphate (TCP) with a solubilization index of 1.62. The concentration of soluble phosphorus (P) in the culture medium increased (up to 66.44 μg mL^-1^) with concomitant decrease in pH (up to 4.3) after 10 days of bacterial growth (**Figure 1A**). A time-course HPLC analyses of cell-free supernatant showed the production of gluconic acid (GA) and malic acid (MA) by KPS-11 (**Figure 1B**). The production of both acids increased with time but GA was produced in higher amount as compared to MA (**Figure 1B**). A positive correlation (*p* < 0.001) was observed among decrease in pH, acid production and P-solubilization (*r* = 0.857). Similarly, production of GA and MA were positively correlated (*p* < 0.001) with pH decrease (*r* = 0.944, 0.836 respectively).

### Phytase Activity and Detection of Phytase Encoding β-Propeller Gene

**FIGURE 2:**
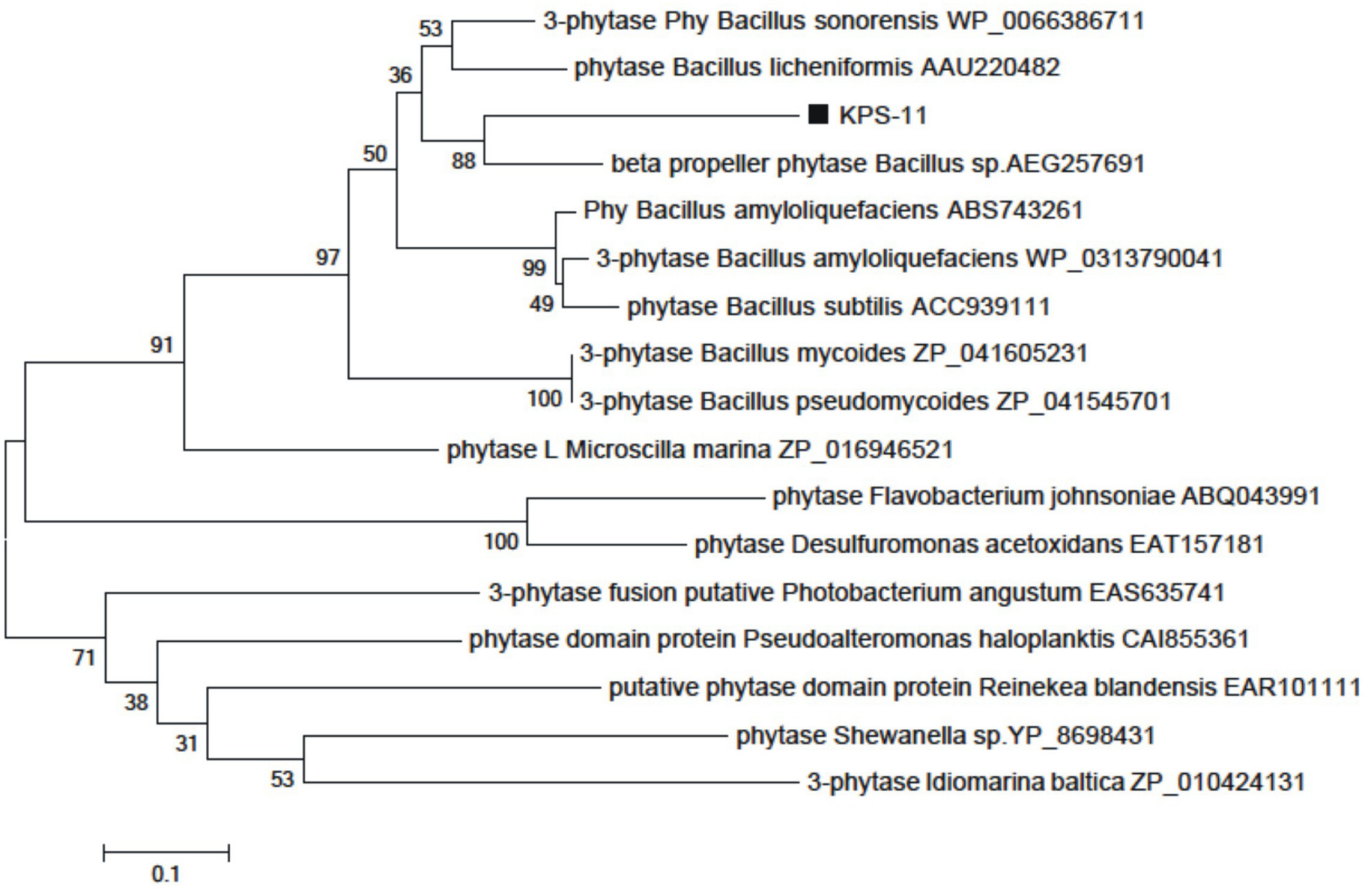
**Phylogenetic tree generated by the Neighbor-Joining method based on full-length amino acid sequences of BPPs**. The position of *B. subtilis* strain KPS-11 phytase is shown relative to other BPPs. The microbial source, Genbank accession numbers of the BPPs are shown. Bootstrap values are expressed as percentages of 1000 replications.

**FIGURE 3:**
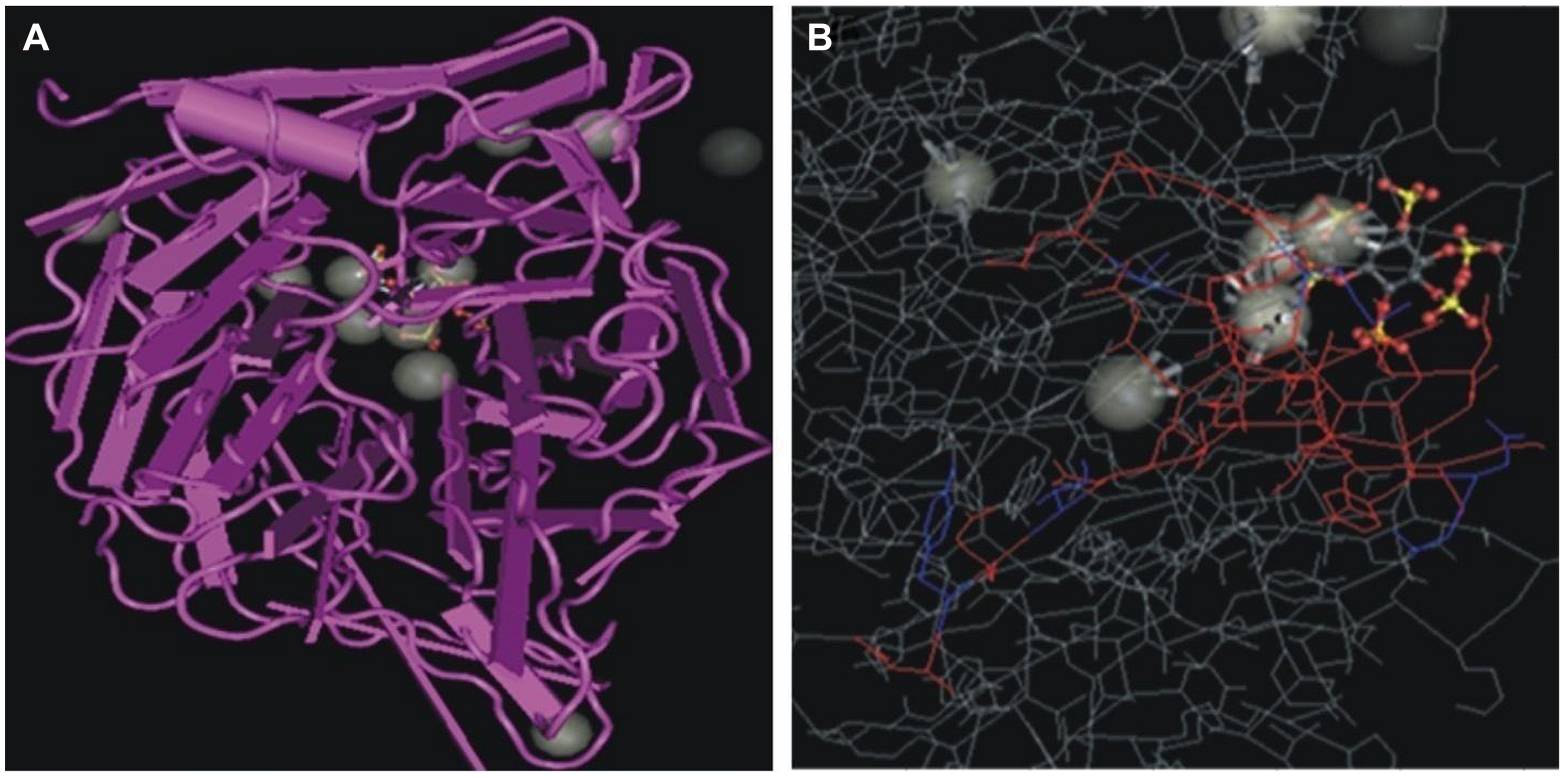
**(A)** Three-dimensional structure of BPP from *B. subtilis* (MMDB ID 89811). **(B)** BPP structure of *B. subtilis* strain KPS-11 (colored) in comparison with BPP of *B. subtilis* (NCBI PDB ID: 3AMR) visualized using Cn3D software.

*Bacillus subtilis* strain KPS-11 mineralized Na-phytate and produced 4 cm clear halo zone on phytase specific agar medium. The phytase activity assays revealed that *B. subtilis* strain KPS-11 showed both extracellular and cell associated phytase enzyme activity. The results showed that there was comparatively higher extracellular 4.3 × 10^-10^ kat mg^-1^ phytase activity than cell associated 1.6 × 10^-10^ kat mg^-1^. Sequence homology searches of amino acids from translated nucleotides showed that phytase gene shares 92% similarity with 3-phytase gene from *B. subtilis* (Genbank accession no.WP_014114128.1). An alignment with other BPPs (phytase encoding genes) was performed using the Clustal W program, and a phylogenetic tree was constructed based on the alignment using the Neighbor-Joining method (**Figure 2**). The tree topology shows that BPP gene of KPS-11 is phylogenetically related to other BPP genes reported from *Bacillus* species. The MMDB from NCBI combined with the software Cn3D 4.3 revealed that amplified phytase is within the same phytase- superfamily. 3D structures of amplified gene showed 83% similarity to the calcium-dependent BPP described for *B*. *subtilis* (NCBI PDB ID 3AMR; **Figure 3**). Furthermore, the gene shows the presence a putative signal peptide at position 1–21 when analyzed on SignalP.

### Green House Potato Inoculation and Colonization Experiment

**FIGURE 4:**
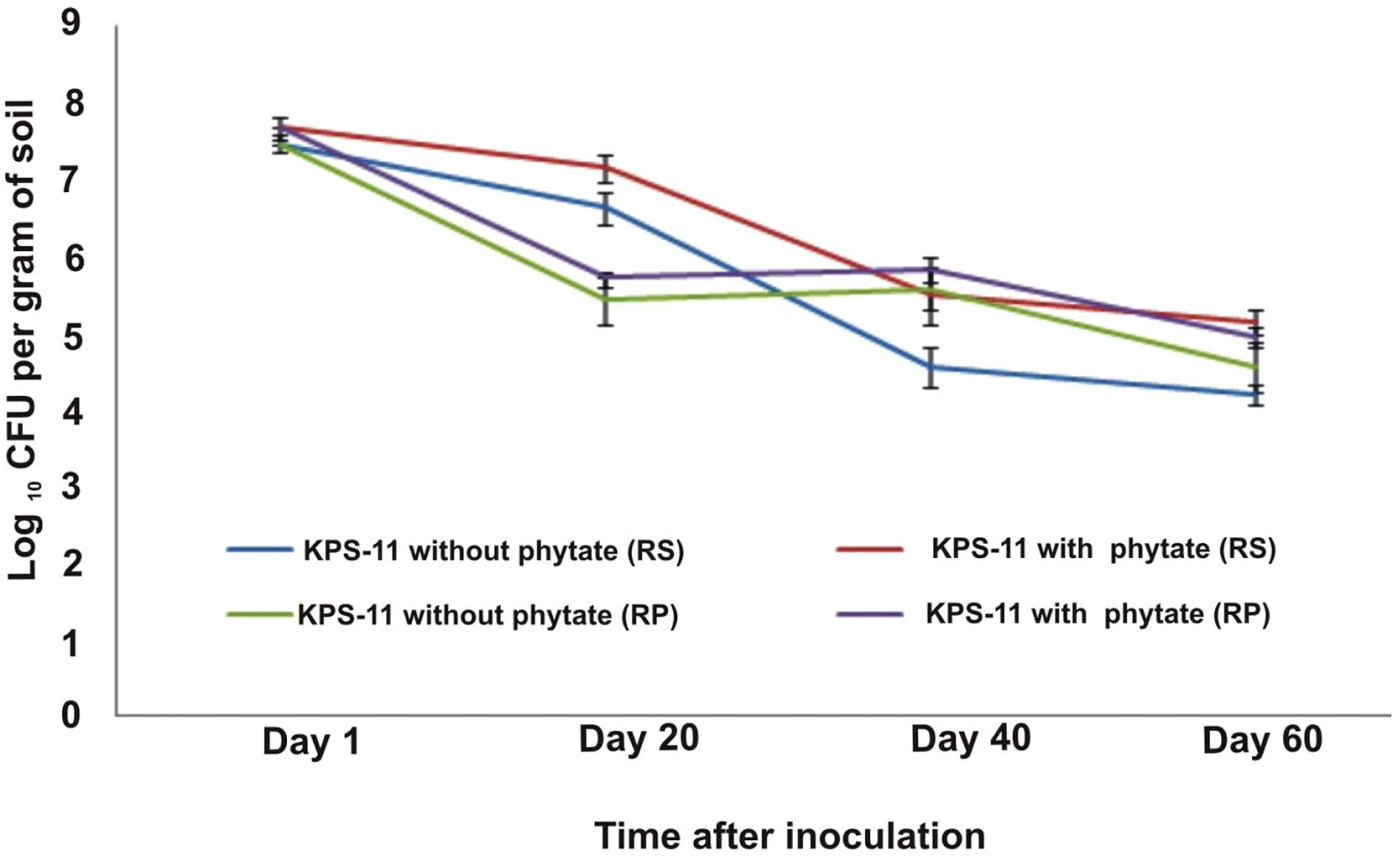
**Colonization and survival of rifampicin-resistant derivatives of *B. subtilis* strain KPS-11 with potato rhizosphere and rhizoplane**. The CFU of derivatives of KPS-11 were recovered both from rhizosphere and rhizoplane with standard procedure and converted to log values and plotted against time. No population was determined in un-inoculated control on LB-rifampicin agar.

Rifampicin resistant derivative were develop by growing *B. subtilis* strain KPS-11 on LB plates containing antibiotic concentration of 70 μg mL^-1^. Resistant derivative were selected for studying its survival in potato rhizosphere and rhizoplane and plant growth promotion. A change in bacterial population was observed in rhizosphere and rhizoplane from 20 to 60 DAS (**Figure 4**). Bacterial populations in KPS-11 inoculated soils supplemented with or without Na-phytate (substrate) were high (7.66 and 7.45 cfu g^-1^ soil, respectively) after 3 h of inoculation. In potato rhizosphere, after 20 DAS it was observed that in KPS-11 inoculated soils supplemented with or without Na-phytate, bacterial population decreased (7.13 and 6.62 cfu g^-1^ soil, respectively) and continued to decrease upto 40 DAS (5.13 and 4.19 cfu g^-1^ soil) after which population became constant at 60 DAS (5.48 and 4.55 cfu g^-1^ of rhizosphere soil).

On the other hand, rhizoplane bacterial populations in inoculated soils either supplemented with or without substrate first showed a considerable decrease at 20 DAS (5.70 and 5.41 cfu g^-1^ root fresh wt., respectively); a slight increase at 40 DAS (5.83 and 5.56 cfu g^-1^ root fresh wt.) and a significant decrease at 60 DAS (4.93 and 4.54 cfu g^-1^ root fresh wt.). No bacterial population was observed (on LB plates containing rifampicin) from un-inoculated control soil (**Figure 4**).

**FIGURE 5:**
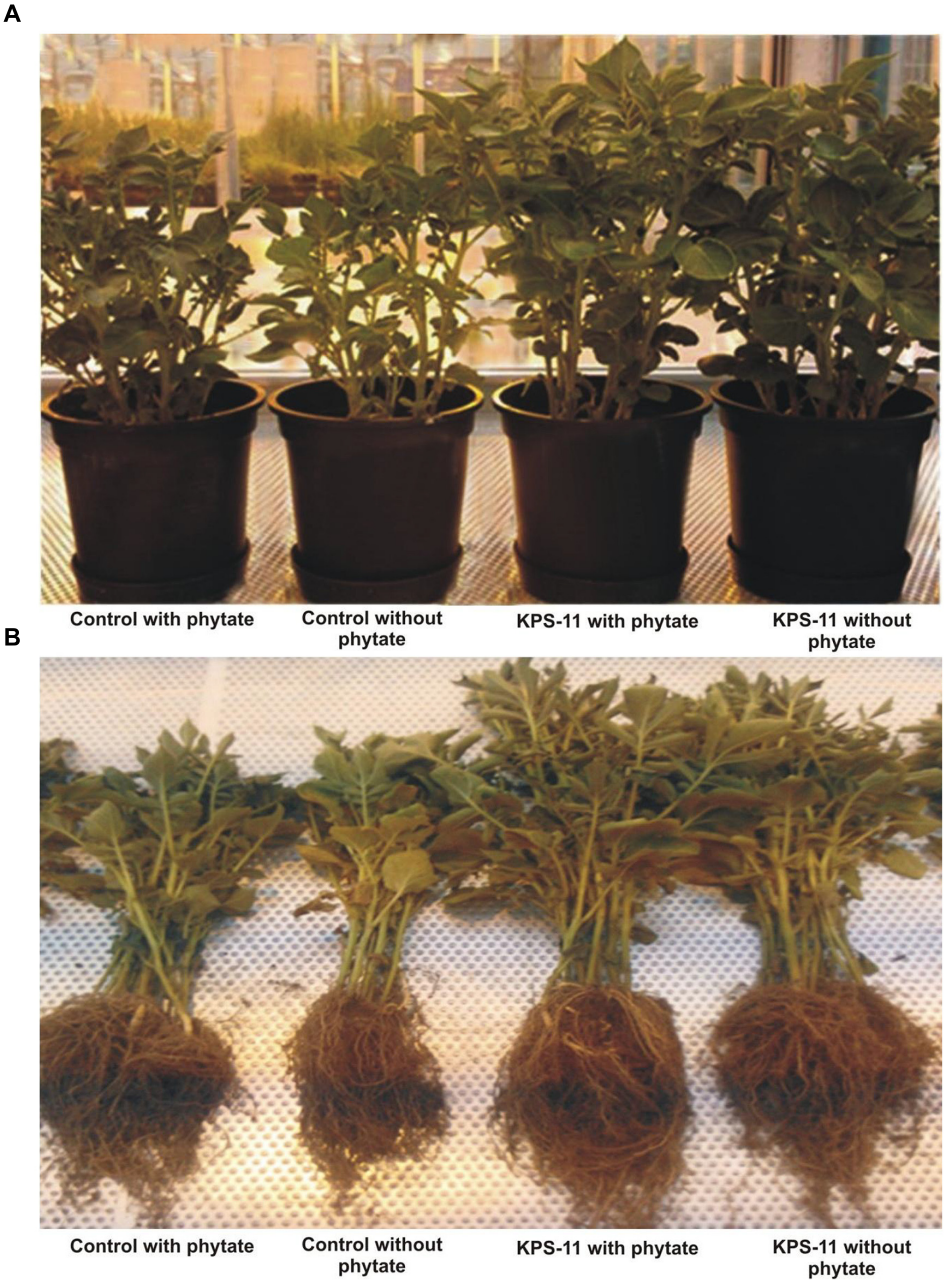
**Representation of above ground (shoot) in a pot view **(A)** and below ground (harvested root) **(B)** biomass of potato cultivar Innovator at 60 days after inoculation with *B. subtilis* strain KPS-11; Co, Zero control, un-inoculated without substrate (Na-phytate); Cs, un-inoculated with substrate; No, inoculated without substrate; Ns, inoculated with substrate**. (*Innovator is a variety of Nederland’s Potato Consultative Foundation (NIVAP), Netherlands and SASA, UK*).

**FIGURE 6:**
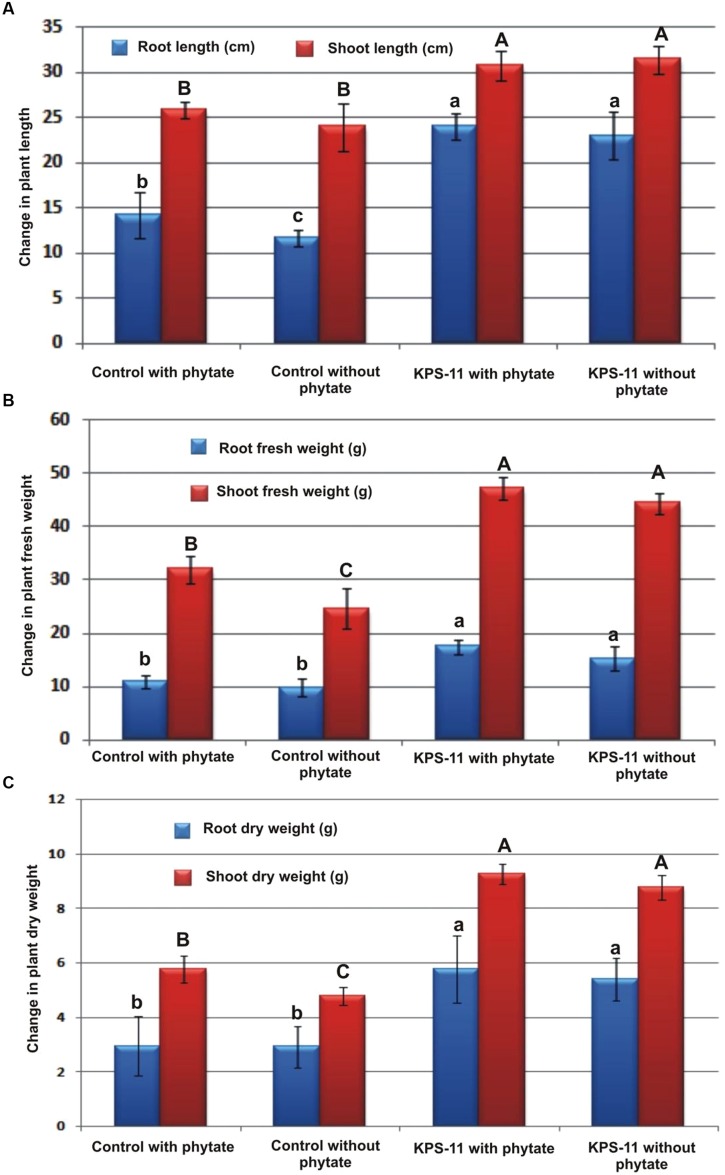
**Effect of inoculation with *B. subtilis* strain KPS-11 on different growth parameters of potato cultivar Innovator**. The data represents **(A)** root length and shoot length, **(B)** root and shoot fresh weight, **(C)** root and shoot dry weight. Column bar shares same letter(s) for each parameter do not differ significantly at *p* = 0.05. Error bars shows standard deviation of data. The data is mean of four replicates. (*Innovator is a variety of Nederland’s Potato Consultative Foundation (NIVAP), Netherlands and SASA, UK).*

Observations about growth parameters recorded at 60 DAS revealed Statistically significant increase (*p* ≥ 0.05 for all) in root and shoot length, and root and shoot fresh and dry weight for inoculated plants grown in soil supplemented with substrate as compare to un-inoculated plants grown in soil supplemented with or without substrate (**Figures 5** and **6**). KPS-11-inoculated plants in soil supplemented with or without substrate also showed significant increase (*p* ≥ 0.05) in root, shoot length and fresh and dry weight as compared to un-inoculated (controls) plants. KPS-11-inoculated plants in soil without substrate showed 95.75 and 31.25%, increase in root and shoot length and 72.49, 80.27% (root and shoot fresh weight), 84.35, 82.32% (root and shoot dry weight) over un-inoculated plants in soil without Na-phytate. Similarly, KPS-11-inoculated plants in soil containing substrate showed 20.89 and 19.18% increase in root and shoot length and 52.15, 46.97% (root and shoot fresh weight), 95.94, 60.83% (root and shoot dry weight) as compare to un-inoculated plants in soil containing Na-phytate. Un-inoculated plants in soil containing Na-phytate as substrate showed significant increase for root length, shoot fresh and dry weight as compared to un-inoculated plants in soil without substrate.

## Discussion

Bioengineering and rhizosphere management for efficient P-utilization in crops is of prime importance in agriculture these days due to rapidly increasing cost of fertilizers as well as environmental concerns of high fertilizer usage especially in edible crops. The bacteria having the ability of both phosphate-solubilization and phytate-mineralization are widespread in the rhizosphere of different crops (Unno et al., 2005; Jorquera et al., 2008b; Shahid et al., 2012) and offer great promise to agricultural applications because they increase the level of soluble P in soil and facilitate its uptake by plants (Konietzny and Greiner, 2004; Richardson and Simpson, 2011).

We have isolated and characterized a bacterium KPS-11 from potato rhizosphere with the potential to solubilize inorganic phosphate and mineralize organic phosphate *in vitro.* The bacterium identified as *B. subtilis* was able to produce 6.48 μg mL^-1^ IAA in tryptophan supplemented medium, utilized 40 different carbon sources and showed resistance against 20 different chemicals. This shows that *B. subtilis* KPS-11 is metabolically versatile and well-adapted. IAA is a plant growth regulating hormone that increases root development and induces root proliferation in plants (Shahid et al., 2012; Imran et al., 2014). KPS-11 was able to solubilize 66.44 μg mL^-1^ tri-calcium phosphate within 10 days P-solubilization. The range of inorganic P-solubilization by KPS-11 is almost similar as reported earlier (Oliveira et al., 2009; Qian et al., 2010). Moreover, it showed both extracellular as well as cell-associated phytase activity (4.3 × 10^-10^, 1.6 × 10^-10^ kat mg^-1^, respectively) *in vitro*. Phytohormone production such as IAA is an additional trait along with other plant growth promoting attributes that can also improve the P acquisition indirectly by increasing plant root growth (Acuña et al., 2011; Marschner et al., 2011). IAA has been implicated in plant growth promotion by lateral root development, along with phosphate solubilizing potential (Seo and Park, 2009; Bianco and Defez, 2010; Venieraki et al., 2011).

The HPLC analysis of culture supernatant showed the production of gluconic acid (17.32 μg mL^-1^) and malic acid (5.15 μg mL^-1^) with significant decrease in pH of the medium (from 7 to 4.3) within 10 days. The potential and ability to release metabolites particularly organic acids is the main phosphate solubilizing mechanism in many bacteria. These metabolites carry hydroxyl and carboxyl groups that chelate the phosphate bound cation and coverts it to soluble form (Rodriguez et al., 2004; Qian et al., 2010). Both phosphate solubilization and organic acid production (GA, MA) were found directly proportional to time of incubation and showed an increase with the passage of time as described earlier (Chen et al., 2006; Shahid et al., 2012) and were found positively correlated with each other (*r* = 0.857, 0.944, 0.836 respectively).

A group of *pqq* genes (*pqq*ABCDEF) is responsible for encoding proteins (polypeptides), precursors for PQQ synthesis. PQQ is considered as co-factor in extracellular oxidation of glucose to gluconic acid in the presence of GDH. Universal primers *pqq*F/pqqR^2^ (Perez et al., 2007) were used for the amplification of the *pqq*E gene in bacterial strain KPS-11 using conditions described (Shahid et al., 2012) in repeated PCR experiments but we could not get any amplification. This shows that although the organic acids are produced by this strain and insoluble P is being solubilized but the genes might be different from those reported earlier and some other primers targeting different portion of the gene may be used to amplify the pqq gene.

Soil organic P is mainly hydrolyzed by phytases (Bünemann, 2008) and among four described classes of phytases so for, BPP is considered to be the major class of phytate-degrading enzymes in nature and appear to be the predominant phytase in *Bacillus* species (Greiner et al., 2007; Huang et al., 2009). In current study, in relation to phytase characterization, the BLASTP results showed that the phytase-encoding (BPP) gene found in KPS-11 was 92% similar to 3-phytase (3.1.3.8) from *B. subtilis* and 3D structure analysis revealed 83% similarities to alkaline-phytase described by Zeng et al. (2011). As a major phytate-degrading class of phytases particularly under neutral to alkaline conditions in the presence of Ca^2+^ (Cheng and Lim, 2006), BPP containing rhizobacteria have great potential as inoculant for soils with pH neutral to calcareous like most of the soils in Pakistan.

In green house study, potato inoculation with BPP containing strain KPS-11 showed potential to increase root, shoot length and also fresh and dry weight significantly mainly in pots supplemented with substrate (Na-phytate) as compared to un-inoculated plants. The likely reason may be the KPS-11 mediated conversion of organic P substrate added to soil which was rapidly hydrolyzed in response to phosphatases activity of KPS-11 (Lim et al., 2007; Richardson and Simpson, 2011). Moreover, addition of phytate might have increased the copy number of *Bacillus* BPP gene in the rhizosphere (Jorquera et al., 2013). Analysis of bacterial population dynamics showed that in rhizosphere for both phytate-supplemented and non-supplemented plants, generally the population density initially decreased and then attains a constant level. These findings corroborate earlier report by Fischer et al. (2010) for wheat rhizosphere and Andreote et al. (2009) for potato rhizosphere. There may be more than one reason for fluctuations in bacterial population density but obviously plant growth stage is the strongest factor that affects the indigenous plant associated communities in field grown potato plants (Van Overbeek and Van Elsas, 2008). KPS-11 population was higher in phytate-supplemented pots as compared to non-supplemented because phyate act as ‘hot spot’ for the bacteria containing BPP. The potential of KPS-11 to solubilize inorganic P-solubilization *in vivo* by applying inorganic P in pots needs to be elucidated alongwith the amplification of pqqE gene of KPS-11 using different primers.

Furthermore, presence of KPS-11 in the rhizosphere of both phytate-supplemented and non-supplemented plants shows its rhizosphere competence and colonization potential. Rhizosphere competence and colonization are much desired characters for biofertilizer production. Number of plant growth promoting rhizobacteria (PGPR) usually not high enough to compete with other indigenous bacteria commonly present in the rhizosphere, therefore, root colonization is an important trait of bacteria and it can be strain-specific. Due to poor colonization or out-competition by native rhizosphere bacteria sometimes, rhizobacteria screened for their beneficial plant growth promoting attributes *in vitro* fail to perform *in vivo* (Benizri et al., 2001).

Phosphobacteria having multiple PGPR traits like phytohormones needs a lot to be desired (Raddadi et al., 2008; Acuña et al., 2011) and member of genus *Bacillus* have been well documented for multifaceted plant-beneficial activities (McSpadden Gardener, 2004; Choudhary and Johri, 2009). These multiple PGPR traits may act synergistically e.g., phytohormone production also improve the P acquisition indirectly by increasing plant root growth (Acuña et al., 2011; Marschner et al., 2011) and lateral root development (Seo and Park, 2009; Bianco and Defez, 2010; Venieraki et al., 2011). Biofertilizer based on single strain having multiple traits is more cost-effective and user-friendly as compared to handling a combination of PGPR with having single trait each.

## Conclusion

The bacterial strain named KPS-11 was isolated from potato field soils of central Punjab, Pakistan that typically is well known potato growing region. This bacterium identified as *B. subtilis* by 16S *rRNA* sequence analysis proved an efficient PGPR having multiple traits including mineralization of organic phosphate, solubilization of inorganic phosphate, and production of plant growth promoting hormone IAA. Alongwith multifaceted beneficial characteristics *in vitro*, KPS-11 exhibited potato growth promotion and colonization potential *in vivo*. Based on these facts, KPS-11 represents itself as a potential candidate for use in integrated nutrient management strategies for sustainable potato cultivation in Pakistan. This bacterium may further be explored for its applications in different crops at global level.

## Conflict of Interest Statement

The authors declare that the research was conducted in the absence of any commercial or financial relationships that could be construed as a potential conflict of interest.
